# Leveraging Artificial Intelligence in Advance Care Planning: A Scoping Review

**DOI:** 10.1093/geroni/igaf122.4153

**Published:** 2025-12-31

**Authors:** Minghui Tan, Jinfeng Ding, Siyuan Tang

**Affiliations:** Central South University, union city, New Jersey, United States; Central South University, Changsha, Hunan, China (People’s Republic); Central South University, Changsha, Hunan, China (People’s Republic)

## Abstract

Background Advance care planning (ACP) is a process that enables individuals to discuss future health care decisions before they become seriously ill or unable to communicate. Artificial intelligence (AI) has demonstrated promising outcomes in facilitating healthcare, offering the potential to facilitate ACP. However, the current status of using AI to facilitate ACP is unclear. This study aimed to investigate how AI has been leveraged to facilitate ACP, with a particular focus on the intended purposes, AI algorithms used, data sources, and the performance of AI in achieving the intended purposes. Methods The methodology employed in this study adhered to the Scoping Review Methodological Framework. PubMed, EMBASE, Web of Science, CINAHL, Cochrane Library, and IEEE Xplore databases were searched from their inception to July 2025. Descriptive analysis and narrative synthesis were used to summarize findings from the included studies. Results A total of 42 eligible studies was analyzed. The studies were primarily used to detect ACP conversations and documents, identify patients needing ACP, promote ACP education, and explore linguistic features in ACP conversations. Rule-based natural language processing emerged as the most commonly used AI algorithm, with textual data being the primary modality employed. The included studies exhibited significant variation in performance evaluation. Finding The current use of AI in ACP remains limited in scope, primarily focused on extracting ACP documentation from electronic health records and identifying patients who may benefit from ACP. The use of advanced technologies such as generative AI is limited, and performance evaluation primarily relied on discrimination metrics.

